# FORGING COMMUNITY–ACADEMIC PARTNERSHIPS FOR HEALTHY AGING: A COMMUNITY-LED TOOLKIT TO COMBAT AGEISM

**DOI:** 10.1093/geroni/igad104.2505

**Published:** 2023-12-21

**Authors:** Dana Urbanski, Elma Johnson, Maby Almiron, Steve Chapman, Amina Keinan, Phyllis Thomas, Mayla Yang, Robbin Frazier

**Affiliations:** University of Minnesota, Minneapolis, Minnesota, United States; University of Minnesota, Minneapolis, Minnesota, United States; Center for Healthy Aging and Innovation, University of Minnesota, Minneapolis, Minnesota, United States; Center for Healthy Aging and Innovation, University of Minnesota, Minneapolis, Minnesota, United States; Center for Healthy Aging and Innovation, University of Minnesota, Minneapolis, Minnesota, United States; Center for Healthy Aging and Innovation, University of Minnesota, Minneapolis, Minnesota, United States; Center for Healthy Aging and Innovation, University of Minnesota, Minneapolis, Minnesota, United States; University of Minnesota, Center for Healthy Aging and Innovation, Minneapolis, Minnesota, United States

## Abstract

There is growing recognition that community-engaged research approaches are essential for bridging the evidence-to-practice gap, especially for minoritized communities underrepresented among academic researchers. Such approaches often adopt a consultative model in which community members provide insights, perspectives, and feedback to support academic-driven projects. This strategy can be mutually beneficial when paired with cultural sensitivity and humility; however, there is a pressing need to form community-academic partnerships that flip this consultative relationship—placing university researchers as advisers on community-led projects that reflect local needs, priorities, and values. Here, we highlight the work of one such community-academic partnership led by the Center for Healthy Aging and Innovation’s (CHAI’s) Community Advisory Board with support from University of Minnesota faculty, staff, and graduate students. First, we describe the vision and formation of our Community Advisory Board (CAB), along with recruitment of its diverse membership representing eight cultural communities of the Twin Cities. We then outline the collaborative needs assessment process by which the CAB identified and prioritized action areas to advance healthy aging and health equity. Finally, we present preliminary results and early lessons learned from the CAB’s first project initiative—creation of a community-led, culturally responsive toolkit to address and reframe community-based sources of age-related stigma and discrimination. Findings from this project: 1) offer a plausible model and process for community-academic engagement which may be adapted and refined to fit local contexts; 2) underscore the value of community-led academic partnerships for addressing age-related stigma and other social factors associated with poor aging outcomes.

